# Citrus Leaf Volatiles as Affected by Developmental Stage and Genetic Type

**DOI:** 10.3390/ijms140917744

**Published:** 2013-08-29

**Authors:** Muhammad Azam, Qian Jiang, Bo Zhang, Changjie Xu, Kunsong Chen

**Affiliations:** Laboratory of Fruit Quality Biology/The State Agriculture Ministry Laboratory of Horticultural Plant Growth, Development and Quality Improvement, Zhejiang University, Zijingang Campus, Hangzhou 310058, China; E-Mails: azam@zju.edu.cn (M.A.); twinssky1987@163.com (Q.J.); bozhang@zju.edu.cn (B.Z.); akun@zju.edu.cn (K.C.)

**Keywords:** volatiles, young leaf, mature leaf, citrus types, GC-MS

## Abstract

Major volatiles from young and mature leaves of different citrus types were analyzed by headspace-solid phase microextraction (HS-SPME)-GC-MS. A total of 123 components were identified form nine citrus cultivars, including nine aldehydes, 19 monoterpene hydrocarbons, 27 oxygenated monoterpenes, 43 sesquiterpene hydrocarbons, eight oxygenated sesquiterpenes, two ketones, six esters and nine miscellaneous. Young leaves produced higher amounts of volatiles than mature leaves in most cultivars. The percentage of aldehyde and monoterpene hydrocarbons increased, whilst oxygenated monoterpenes and sesquiterpenes compounds decreased during leaf development. Linalool was the most abundant compound in young leaves, whereas limonene was the chief component in mature ones. Notably, linalool content decreased, while limonene increased, during leaf development in most cultivars. Leaf volatiles were also affected by genetic types. A most abundant volatile in one or several genotypes can be absent in another one(s), such as limonene in young leaves of lemon *vs.* Satsuma mandarin and β-terpinene in mature leaves of three genotypes *vs.* the other four. Compositional data was subjected to multivariate statistical analysis, and variations in leaf volatiles were identified and clustered into six groups. This research determining the relationship between production of major volatiles from different citrus varieties and leaf stages could be of use for industrial and culinary purposes.

## 1. Introduction

Terpenoids are probably the most widespread group of volatile secondary metabolites produced by plants [1]. They are derived from two independent pathways leading to the formation of isopentenyl diphosphate (IPP) and dimethylallyl diphosphate (DMAPP), the two basic building blocks. Monoterpenes are C_10_ compounds synthesized from geranyl diphosphate (GPP) via the MEP (2C-methyl-d-erythritol 4-phosphate) pathway in plastids, while sesquiterpenoids (C_15_) are produced from farnesyl diphosphate (FPP) via the mevalonate (MVA) pathway in the cytosol [2–5]. A small group of volatiles and their profiles are responsible for unique flavors for individual food [6]. Different plants and their different tissues emit unique aromas, scents, flavors and fragrances, due to the presence of one or, in most cases, a mixture of several volatile compounds [7,8].

Citrus belongs to a large family, Rutaceae, members of which are cultivated worldwide as fruit crops. Aroma compounds are important for citrus, not only as a critical attribute of fruit quality, but also as valuable commercial products used extensively in cosmetics, food and household items for fragrance [9,10], as well as for medicinal purposes, such as prevention of tumor growth [11] and Gram-positive bacterial infection [12]. Citrus leaves have increasingly been used in volatile studies, because of their richness in volatile compounds, rapid growth and large biomass, and because they are available throughout the year [13], they have been used for characterizing citrus cultivars and also for evaluating the impact of the environment on volatiles [13,14]. Leaf oil composition is more diverse than in fruit and is not over dominated by limonene or limonene/γ-terpinene, which commonly constitute over 70% of total volatiles in fruit peel [15–17]. In general, however, the number of citrus leaf volatile studies is limited; in particular, there is a lack of information for the comparative study of young and mature leaf volatiles from different citrus cultivars. Although volatile changes during the opening of leaf buds and development from young to mature leaves have been reported previously, the study was limited to grapefruit and lemon only [18,19].

Historically, leaf volatiles were analyzed by hydrodistillation [14,20–23] and solvent extraction [24], which takes a long time for analysis. Recently, solid phase microextraction (SPME) integrated with GC-MS has been shown to be much more sensitive, reproducible and efficient for metabolomics studies of volatiles, has been widely used in plant research [25,26], including some studies on citrus fruit volatiles analysis [27–29], and has become established as a suitable substitute for gas chromatography analysis of essential oils. However, to our knowledge, there is only one study of citrus fresh leaf volatile profiling with SPME coupled with GC-MS [30], and this utilized polydimethylsiloxane (PDMS) fiber, rather than a complex fiber mixture of divinylbenzene/carboxen/polydimethylsiloxane (DVB/CAR/PDMS), which is more widely used, because of its ability to extract a larger number of volatiles, rather than other fibers [27,31,32].

In this study, by using HS-SPME coupled with GC-MS and application of a DVB/CAR/PDMS complex fiber mixture, volatile profiles of young and mature leaves of nine cultivars from five citrus types were characterized. The study revealed that monoterpenes are the major components from the leaves of nine citrus cultivars, and differences among cultivars, as well as two developmental stages were observed.

## 2. Results and Discussion

### 2.1. Analysis of Citrus Leaf Volatiles by HS-SPME-GC-MS

In the present study, profiling of young and mature leaf volatiles of nine citrus cultivars from five types (Table 1) was investigated using HS-SPME-GC-MS. The percentage and retention time of the identified compounds are listed in Table 2. A total of 123 compounds were detected and separated on the basis of their chemical structures and were grouped into nine classes, *i.e.*, nine aldehydes, 19 monoterpene hydrocarbons, 27 oxygenated monoterpenes, 43 sesquiterpene hydrocarbons, eight oxygenated sesquiterpenes, two ketones, six esters and nine miscellaneous (Table S1). Forty-eight compounds (three aldehydes, one monoterpene hydrocarbons, 12 oxygenated monoterpenes, 18 sesquiterpene hydrocarbons, two oxygenated sesquiterpenes, two ketones, three esters, two aromatic hydrocarbons, two phenolics, one alcohol and two unclassified) out of 123 were identified for the first time from citrus leaves (Table S1), while the remaining volatiles have been described previously in citrus leaf oil [13,14,18–20,22,30,33].

Most of the newly identified compounds were present in low amounts; nevertheless, 12 major ones, which accounting for over 1% of total volatiles, were observed in at least one citrus leaf sample. In particular, (Z)-α-bergamotene, γ-elemene, myrtenyl acetate and α-farnesene accounted for over 4% of total volatiles in some samples. This might be due to the more advanced analysis techniques or the greater number of citrus cultivars in this study.

Currently, over 300 volatiles have been reported in citrus fruit [34]. Comparison of the leaf volatiles identified in this study with those from flowers and fruit showed that 104 leaf volatiles have been tentatively reported in other citrus volatile studies [16,26–28,31,33,35,36], while the other 19 were reported in other plants, such as myrtenyl acetate in myrtle (*Myrtus comunis* L.) [37], (Z)-α-bergamotene from the essential oil of mulberry [38], *cis*-β-terpineol from *Myrtus* species [39] and pinocarvone from the walnut tree [40]. In addition, the high percentage of aromatic hydrocarbon, *p*-Cymene (14.35%), was only found in young leaf of Satsuma mandarin (Table 2), which has also been reported previously in *C. unshiu* cultivars [41].

### 2.2. Variation in Total Volatiles Content and Major Chemical Classes from Young and Mature Leaf

**Figure 1:**
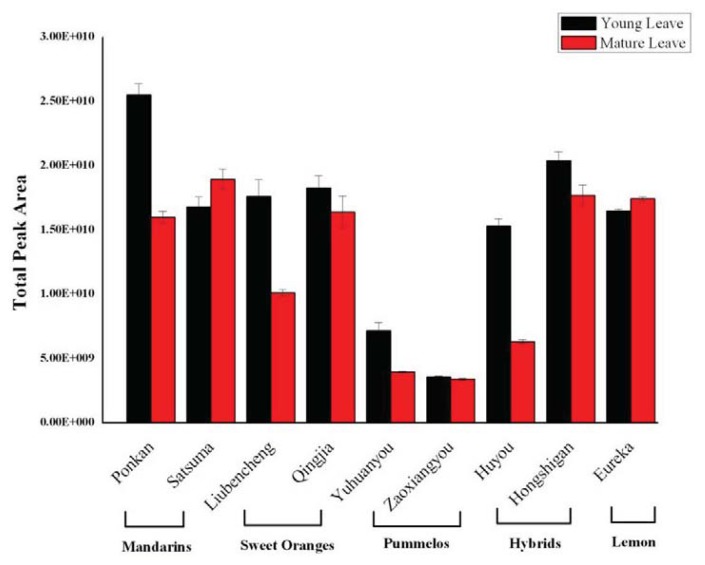
Total volatile contents of young and mature leaves of five citrus types.

Differences in total amount of leaf volatiles, measured by GC-MS total ion current peak area, were observed between citrus types and, to a lesser extent, between cultivars, as well as between leaf developmental stage, especially in Ponkan, Liubencheng, Yuhuanyou and Huyou (Figure 1). The lowest amount of volatiles was found in either young or mature leaves of pomelos, and there was no marked difference between the other cultivars (Figure 1), except that, in general, young leaves produced higher amounts of volatiles than mature leaves (Figure 1).

**Figure 2:**
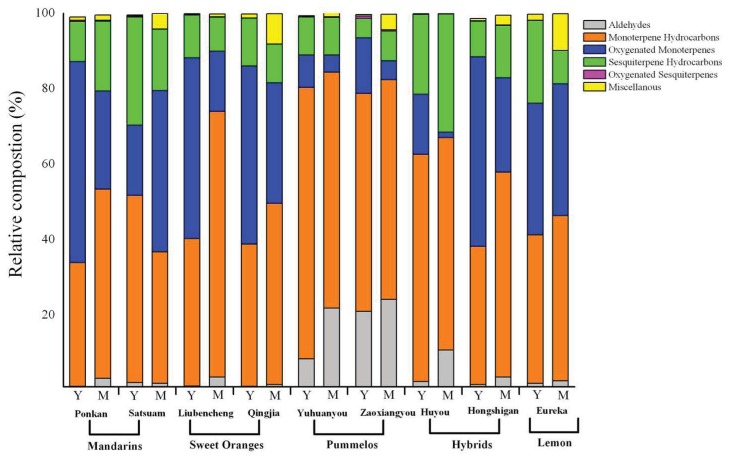
Relative composition (%) of major chemical groups from young (Y) and mature (M) leaves of five citrus types: mandarins (Ponkan, Satsuma), sweet oranges (Liubencheng, Qingjia), pomelos (Yuhuanyou, Zaoxiangyou), hybrids (Huyou, Hongshigan) and lemon (Eureka).

Terpenoids constitute the main part of leaf volatiles, accounting for 71.22 to 98.47% of total volatile amount, and monoterpenes, or monoterpene hydrocarbons and oxygenated monoterpenes together, were the main terpenoids, especially in pomelos (Figure 2). Oxygenated sesquiterpenes were present in a low amount, with a maximum of 0.63% in total volatiles of Zaoxiangyou young leaves (Figure 2). Aldehydes varied greatly among different samples, from 1.60% of total volatiles in Ponkan young leaves to 24.21% in Zaoxiangyou mature leaves (Figure 2). The percentage of each chemical class in total volatiles varied among citrus types and their cultivars. For example, pomelos were rich in monoterpene hydrocarbons (57.74% to 71.93%), but low in oxygenated monoterpenes (0.80%–5.10%), while in young leaves of Ponkan and Hongshigan, the amount of monoterpene hydrocarbons was less than that of oxygenated monoterpenes (Figure 2).

The percentage of each chemical class also varied with leaf developmental stage. Monoterpenes were the major class in leaves regardless of stage and cultivar. Monoterpene hydrocarbons were the most represented class, and mature leaves exhibited high amounts during development, while young leaves were characterized by a higher percentage of oxygenated monoterpenes in most cultivars and by a lower percentage of aldehydes (Figure 2). Sesquiterpenes were present in large numbers, but in lower amounts in almost all cultivars. These observations could indicate that the different accumulation patterns of monoterpenes and sesquiterpenes identified in leaf volatiles may be due to differences in biosynthetic pathways and substrate availability in different citrus cultivars and types.

### 2.3. Variation in Volatile Constituents from Nine Citrus Cultivars

As indicated in Table 2, citrus leaf volatiles were characterized by a high percentage of monoterpene hydrocarbons, predominantly consisting of limonene, β-terpinene, γ-terpinene, E-ocimene and β-pinene. The main oxygenated monoterpenes were linalool, β-citral, citronella and α-citral, while other compounds identified in appreciable abundance were α-terpineol and *cis*-geraniol. The aldehydes were comprised of high percentages of 2-hexenal and hexanal. In addition, the major sesquiterpene hydrocarbons were β-elemene, caryophyllene and γ-elemene, while substantial amounts of α-amorphene, β-farnesene and α-farnesene were also accumulated. The principle ester compound from leaf volatiles was nerol acetate and geranyl acetate (moderate to 5.27%), while only trace amounts of ketones, aromatic hydrocarbons, phenolics and alcohols were found (Table 2).

A wide variation in leaf volatile compounds was observed among the different citrus types and cultivars (Table 2). In order to rank the major volatiles, the average percentage of individual volatiles, in young or mature leaves, respectively, was calculated, and the data for the top 20, on average, for all nine citrus, are shown in Tables 3 and 4. For young leaf volatiles, it was found that linalool ranked first, on average, for all samples, as well as for four individual cultivars, but ranked eleventh or thirty-third for two pomelo cultivars; limonene ranked first in Eureka lemon and ranged between fourth to seventh in most cultivars, but was absent in Satsuma mandarin; E-ocimene and β-pinene were identified as the top two constituents from pomelos, but were absent in sweet oranges and Ponkan; and, similarly, β-terpinene ranked second in three cultivars, but was absent in another three (Table 3).

Among the mature leaf volatiles, limonene ranked first, on average, for all samples, being highest in Eureka lemon and ranging to seventh in Ponkan. E-ocimene and β-pinene were again the top two volatiles from pomelos and were absent from sweet oranges and Ponkan, whereas β-pinene ranked first in Hongshigan. β-terpinene was the most abundant in sweet oranges and Ponkan, but was not found in Satsuma mandarin and pomelos (Table 4). Interestingly, differences in accumulation of some volatiles were observed between cultivars from the same group. For example, β-pinene, β-elemene and *p-*cymene were identified from leaf volatiles of Satsuma mandarin, but were absent in Ponkan. Similarly, β-terpinene was observed in Ponkan and Yuhuanyou, but was not found in Satsuma and Zaoxiangyou. γ-terpinene, on the other hand, was high in leaf volatiles of Huyou and Satsuma, but low in pomelos and Eureka. The fact that monoterpene derivatives, such as linalool and limonene, were the main volatiles found to be present at high concentrations in young and mature leaves is perhaps not surprising, since they are known to play an important role as defense metabolites against herbivores and pathogens [19,30,42–44].

Taken together, each cultivar generates a different profile of volatiles that contribute to its distinct aroma attributes (Figure S1). We found linalool, which has already been reported as a major constituent from leaf volatiles of mandarin [21,30,45] and leaf oils of sour orange [41], to be a major compound in young leaves of most cultivars. Limonene, which was predominantly found in lemon, has been reported previously [13,17], as has γ-Terpinene, which is a major component in leaf oil of Satsuma mandarin [20]. α-bisabolene was only identified in Eureka lemon leaves, which has previously been reported in the lemon, citron and lime [24], and methyl thymol ether was only found in leaf volatiles of Ponkan (mandarin), which has been described previously in mandarin leaf essential oil [41].

Some compounds that have commonly been described in leaf essential oil studies, such as 1, 8-cineol, sabinene and β-phellandrene, as well as some alcohols, such as *cis*-*p*-menth-2-en-1-ol, *trans*-*p*-menth-2-en-1-ol [13,14,24,30,46], were absent in our samples. Similarly, methyl *N*-methyl anthranilate was not found by our approach and, also, in the studies of mandarin leaf oils [20], while it has been described as an important constituent of mandarin leaf oil [14,21,23]. The failure to detect some particular volatile constituents in our samples could be due to genetic (cultivar) differences, or pedoclimatic factors, but we also cannot exclude, as an explanation, the differences in extraction techniques previously used for the identification of chemical variability of leaf volatiles from citrus cultivars [13,14,20,24,47].

### 2.4. Changes in Volatile Abundance during Leaf Development

**Figure 3:**
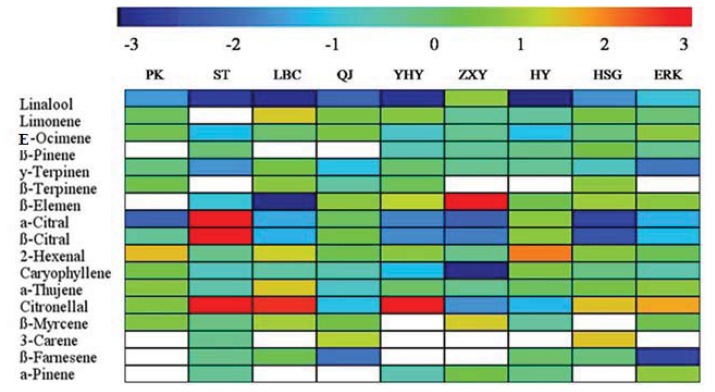
Ratio of volatiles in mature to young leaves from nine citrus cultivars. The full cultivar names corresponding to the abbreviations are as indicated in Table 1. Color code shown above the figure: red shows high, blue low. The blank cells indicate volatiles absent in both young and mature leaves.

Leaf volatiles changed during leaf development, and changes in individual volatiles are listed in both Table 3 and Table 4. The ratio of the content, by percentage, in mature leaf to young leaf was calculated, transformed into log_2_ and is shown in Figure 3. The changes were not consistent among different cultivars. For example, linalool decreased in most cultivars >2-fold, with the exception of Zaoxiangyou, where it increased two-fold; Limonene was found to be >3-fold increased in mature leaves of Satsuma and >1-fold in Liubencheng, but decreased in Zaoxiangyou and Huyou (Figure 3); β-Elemene, on the other hand, decreased three-fold in Liubencheng, but increased by >3-fold in Zaoxiangyou. It was noted that the ratio of α-citral and β-citral was highest in Satsuma, while it decreased in most cultivars and, especially, in Hongshigan (Figure 3). In addition, 2-hexanal increased two-fold in Huyou, yet only slightly increased in other cultivars, while citronellal increased by >3-fold in Yuhuanyou and decreased by >1-fold in Zaoxiangyou.

Our study provides clear evidence that the development phase has an impact on leaf volatile production; some volatiles were increased during the developmental transition from young to mature leaves, while quantitative and qualitative cultivar-specific changes also occurred. It has also been reported previously that the overall emission rate of volatile organic compounds increased from young to older leaves in peppermint, although the qualitative composition of the volatiles changed only slightly [48].

Linalool was found to be higher in young compared to mature leaves for eight out of nine cultivars, as has been reported recently for citrus leaf volatiles [19,30]. A biological role(s) in pollination attraction, protection of reproductive organs from oxidative damage and defense against herbivores and pathogens has been suggested for some volatiles [36,42,49,50].

### 2.5. Multivariate Analysis for Leaf Volatiles among Nine Cultivars

**Figure 4:**
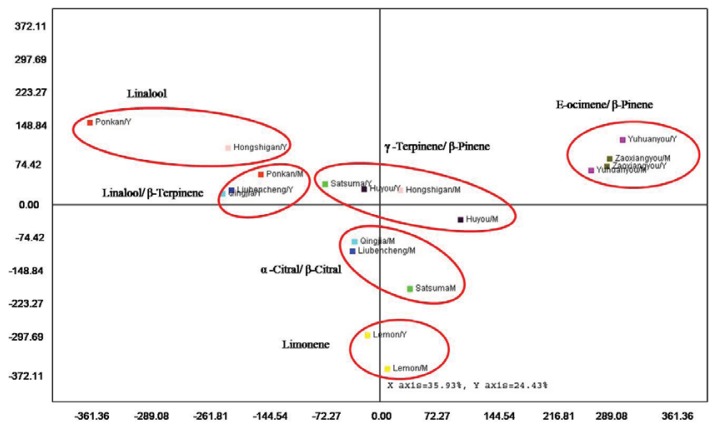
Principle component analysis of both young (Y) and mature (M) leaf volatiles from citrus cultivars.

**Figure 5:**
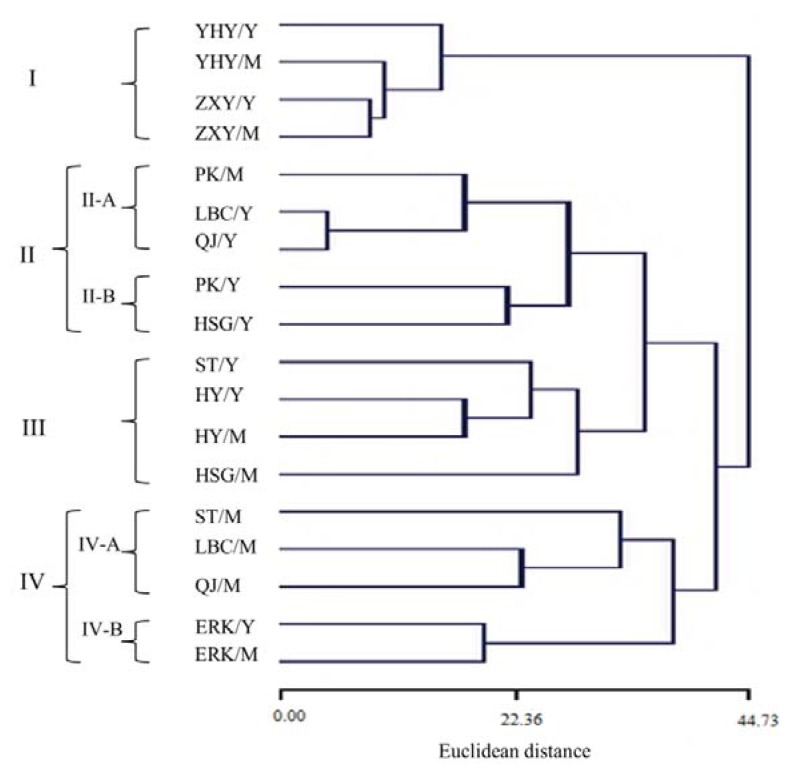
Hierarchical cluster analysis based on Euclidean distance performed on young (Y) and mature (M) leaf volatiles from nine cultivars. Coding names of cultivars: see Table 1.

Multivariate analysis is one of the most appropriate approaches to evaluate variations or diversity of leaf volatiles during leaf development from a range of cultivars. In the present study, the complete data set was selected for principle component analysis (PCA) and hierarchical cluster analysis (HCA) and was performed on average contents of all compounds from each cultivar regardless of developmental stage. The PCA horizontal and vertical axis explained 35.93% and 24.43% of total variance, respectively, and suggested the existence of six clusters from nine cultivars (Figure 4). The dendrogram based on Euclidean distance between cultivars performed on leaf volatiles showed four major groups, with subgroups in groups II and IV (Figure 5).

Group I included volatiles from young and mature leaves of pomelos, with E-ocimene and β-pinene as the major components (Figure 5), which is consistent with the data from PCA analysis (Figure 4). Group II contained two subgroups, with subgroup II-A represented by young leaves of Liubencheng and Qingjia and the mature leaf of Ponkan, comprised of linalool and substantial amounts of β-terpinene, and subgroup II-B, which exhibited a high percentage of linalool in young leaves of Ponkan and Hongshigan only (Figure 5). It was also noted that linalool, the main component of group-II, was present at <2% in all samples of group-I (Table 2). Group III, a γ-terpinene/β-pinene group, was dominated by a high amount of γ-terpinene in young leaves of Satsuma and in Huyou, or β-pinene in mature leaves of Hongshigan (Figure 5). Group IV could be divided into two subgroups, as well, with subgroup IV-A represented by volatiles of mature leaves of Satsuma, Qingjia and Liubencheng, characterized by a high amount of α-citral and β-citral, and subgroup IV-B represented by young and mature leaves of Eureka lemon, with a high percentage of limonene as the major volatile (Figure 5). Notably, the separation of subgroup IV-B (Figure 5) was also observed from PCA analysis (Figure 4).

These results revealed that the concentration of leaf volatiles is variable among citrus types, although an underlying intraspecific similarity and interspecific chemical polymorphism in leaf volatiles was found. A similar finding has been previously reported for the chemical polymorphism of citrus essential oils [22,51]. The segregation of cultivars based on leaf volatile profile is in agreement with phylogenic studies based on morphological and biochemical characteristics (DNA markers) with citron (*C. medica*), mandarin (*C. reticulata*) and pomelo (*C. grandis*), identified as the only true biotypes [52,53]. A chemical variability was observed for Ponkan (*C. reticulata*) and Satsuma mandarin (*C. unshiu*), and similar results have been previously reported [21,22], which emphasized the high chemical polymorphism in mandarins in the Tanaka system [54], which classified mandarins into a different species. Our results, in association with data from previous studies, provide a new insight into leaf volatile variability among cultivars. These results can be helpful for characterization of citrus cultivars based not only on DNA fingerprints, but leaf volatile profiles, as well.

## 3. Experimental Section

### 3.1. Materials

Nine citrus cultivars from 5 types were utilized in this study (Table 1). Leaves were collected in 2011 from adult (10–15 year old) healthy trees, uniform in size and growth vigor, from commercial orchards at Huangyan, Wenzhou and Wenling cities of Zhejiang Province, China. Leaves were sampled from the outer layer of the middle part of the canopy during full blossom, with young leaves, about half the length of full expanded ones, picked from spring flushes of the current year and mature leaves from shoots of the previous year. The samples were stored in dry ice after collection, transferred to the laboratory within 4 h and immediately immersed into liquid nitrogen and kept at −80 °C until analyzed. Three biological replicates were collected for each cultivar from nine plants, with samples from three plants as a biological replicate.

### 3.2. HS-SPME Extraction

One gram of frozen fully ground leaf powder was put into a 10 mL glass vial, and 5 mL of saturated sodium chloride solution were added to stop enzymatic degradation and to help to drive the volatiles into the headspace. Before capping of the vial, 50 μL (0.1%, *v*/*v*) 1-hexanol was added as an internal standard, and then, each headspace was subjected to solid phase micro-extraction (HS-SPME). Extraction was carried out using a 6 mL sample transferred into a 10 mL crimp cap headspace vial. Fifty/thirty micromoles of CAR/DVB/PMDS (Supleco, Bellefonte, PA, USA) fiber were used for the analysis. After incubating the samples at 40 °C for 30 min with continuous agitation (600 rpm), volatile compounds were extracted for 30 min under the same conditions (40 °C, 600 rpm).

### 3.3. GC-MS Analysis

The GC-MS analysis was carried using a 7890A GC gas chromatograph interfaced with a 5957C (inert XL MSD with triple-axis detector) mass spectrometer (Agilent Technologies, Santa Clara, CA, USA). A HP-5MS capillary column (5% Phenyl methyl siloxane, 30 m × 0.25 mm i.d., 0.25 μm film thickness; J & W Scientific, Folsom, CA, USA) was used. The volatiles were desorbed in the GC injection port at 250 °C. The oven temperature was programmed to run at 40 °C for 3 min, then to ramp up to 130 °C at a rate of 3 °C min^−1^ and held for 13 min, again ramped up to 230 °C at a rate of 15 °C min^−1^, and, finally, held for 8 min. Helium was used as the carrier gas at a flow rate of 1 mL/min. The effluent was transferred to the mass detector, and mass spectra were obtained at an ionization energy of 70 eV with the transfer temperature of 250 °C and the source temperature of 230 °C. Data acquisition was performed in scanning mode (mass range *m*/*z* 35–350; 7 scans s^−1^). Chromatogram spectra were recorded and processed using the Enhanced Chemstation software for GC-MS (Agilent G1701EA MSD). *n*-alkanes standards (C7–C40) (SUPELCO-USA) was analyzed, as well, for calculation of retention indices (RI) of citrus volatiles. Identification of volatiles was preliminary based on retention indices (RI) from the literature and related online databases and retention time (RT) with those of the authentic standards available, and further identification was based on matching mass spectral fragmentation patterns with those stored in the NIST/EPA/NIH Mass Spectral Library (NIST-08) of the GC-MS data systems. Relative percentage amounts of the identified compounds were obtained by normalizing the data using the internal standard method.

### 3.4. Electronic Nose Measurements

Leaf volatiles were evaluated by using a FOX 4000 electronic nose (e-nose) (Alpha MOS, Toulouse, France) equipped with 18 metallic oxide sensors according to the methods of [35]. Briefly, one gram of frozen fully ground leaf powder and 5 mL saturated sodium chloride solution, used to drive the volatiles into the headspace, were mixed in a 10 mL tube. Two milliliters of the prepared homogenate were then transferred and sealed in a 10 mL vial, heated at 40 °C for 30 min, and finally, 2 mL of headspace gas were injected into the e-nose for analysis. The signal acquisition lasted for 2 min, followed by 4 min for baseline recovery.

### 3.5. Statistical Analysis

Leaf volatiles were determined from total ion current chromatograms (TIC) generated by GC-MS. The peak areas of all the compounds relative to internal standard (1-hexanol) were used to calculate the percentage of individual volatile, and mean values were used for multivariate analysis. Principle component analysis (PCA) and hierarchical cluster analysis (HCA) analysis were performed with the Euclidean distance and average method (unsupervised clustering method) with the mean percentages [22,23] using the MultiExperiment viewer (MeV_4.8.1) software (http://www.tm4.org, Dana-Farber Cancer Institute, Harvard Medical School, Boston, MA, USA) for analyzing the chemical variability of leaf volatiles among different samples.

## 4. Conclusions

The volatile profiles from young and mature leaves from nine citrus cultivars, analyzed using a high-resolution, sensitive and powerful HS-SPME-GC-MS platform, showed that monoterpenoids were the most abundant compounds. The major components were linalool, limonene, E-ocimene, β-pinene, β-terpinene, γ-terpinene, β-elemene, α-citral and β-citral. Intraspecific similarities and major interspecific chemical polymorphisms were noted between cultivars, but the differences were mainly quantitative, and only a few were cultivar-specific, such as E-ocimene, β-pinene, β-terpinene and *p*-cymene. Multivariate analysis identified the major compounds, revealing interesting relationships between leaf development and cultivars, which further suggested the existence of six major genetic groups. Changes in volatile constituents between young and mature leaf stages suggest some biological roles for these volatiles in, for example, pollination, protection of reproductive organs and defense against herbivores and pathogens. The present work makes a valuable contribution toward determination of the botanical origin of leaf volatiles and, also, enriches the databank of leaf volatiles. In addition, it can contribute to a more complete understanding of the roles of citrus leaf volatiles during plant evolution, development and environment responses. Furthermore, different volatile patterns between cultivars could be used in a wide variety of applications, such as in food, cosmetics, perfumes and medicinal industries.

## Figures and Tables

**Table 1:** Citrus cultivars included in the leaf volatile study.

	Citrus types	Common name	Cultivars	Code
1	*C. reticulata* Blanco	Mandarin	Ponkan	PK
2	*C. unshiu* Marc.	Mandarin	Satsuma	ST
3	*C. sinensis* (L.) Osbeck	Sweet orange	Liubencheng	LBC
4	*C. sinensis* (L.) Osbeck	Sweet orange	Qingjia	QJ
5	*C. grandis* (L.) Osbeck	Pomelo	Yuhuanyou	YHY
6	*C. grandis* (L.) Osbeck	Pomelo	Zaoxiangyou	ZXY
7	*C. changshanensis* Chen et. Fu (*C. aurantium × C. grandis*)	Citrus Hybrid	Huyou	HY
8	*C. reticulate × C. sinensis*	Citrus Hybrid	Hongshigan	HSG
9	*C. limon* (L.) Burm.	Lemon	Eureka	ERK

**Table 2:** Identification of leaf volatiles and quantification of their abundance in young and mature leaves of nine citrus cultivars from five types.

RI a	Compound name						Young leaves								Mature leaves					

		FC c	PK d	ST	LBC	QJ	YHY	ZXY	HY	HSG	ERK	PK	ST	LBC	QJ	YHY	ZXY	HY	HSG	ERK
**Aldehydes**																				
809	Hexanal	A1	M	M	M	M	1.20	4.30	M	M	M	M	M	M	M	6.86	7.05	2.67	M	M
815	2-hexenal	A2	M	1.31	M	M	7.15	13.64	1.74	1.17	1.20	2.21	1.37	2.63	1.25	14.29	14.60	7.83	2.23	1.72
911	Heptanal	A3	/e	/	/	/	/	/	/	/	/	/	/	/	/	/	/	T	/	/
921	2,4-hexadienal, (E,E) *,^b^	A4	T	T	T	T	/	/	T	T	T	T	T	T	/	M	/	M	T	T
978	Benzaldehyde *	A5	/	/	/	T	/	/	/	T	T	/	/	/	/	/	/	/	/	/
1058	Melonal *	A6	/	/	/	/	/	/	/	/	/	/	/	/	/	/	/	/	/	T
1109	Nonanal	A7	M	/	/	/	T	3.00	/	/	/	/	/	/	/	M	2.51	T	/	/
1210	Decanal	A8	T	T	T	T	T	T	T	T	M	M	M	T	T	M	T	/	M	M
1312	Undecanal	A9	/	/	/	/	/	/	/	/	T	/	T	/	/	/	/	/	T	M
**Monoterpene hydrocarbons**																				
922	α-thujene	MH1	2.70	M	2.58	3.27	M	M	M	2.24	T	5.27	M	7.95	2.26	M	M	1.03	3.70	/
945	α-pinene	MH2	/	1.32	/	/	2.56	1.96	2.24	/	M	/	1.33	/	/	2.10	3.11	2.56	/	M
962	camphene	MH3	T	T	T	T	M	M	T	/	/	T	T	T	T	M	M	T	T	/
992	β-pinene	MH4	/	5.97	/	/	27.91	21.32	10.18	11.23	1.04	/	7.29	/	/	21.88	20.65	11.11	18.05	1.06
993	β-terpinene	MH5	14.38	/	15.18	15.36	4.04	/	/	1.65	/	21.83	/	30.55	13.64	5.70	/	M	3.38	/
997	β-myrcene	MH6	1.88	M	2.01	2.17	/	M	1.01	/	1.22	3.50	M	4.74	3.46	/	1.41	M	/	1.80
1011	α-phellandrene	MH7	/	/	/	/	/	/	/	M	M	/	/	/	/	/	/	/	/	/
1013	3-carene	MH8	/	/	4.23	3.31	/	/	/	1.88	/	/	M	/	8.51	/	/	/	5.87	/
1019	α-terpinene	MH9	M	M	M	M	M	M	M	M	/	1.07	M	1.66	M	M	M	M	M	/
1029	*p*-cymene	MH10	/	14.35	/	/	/	/	/	/	/	/	/	/	/	/	/	/	/	/
1030	Limonene	MH11	2.99	/	4.34	4.10	3.64	4.60	7.91	4.63	31.71	4.11	13.71	13.06	7.29	6.93	4.47	7.40	7.78	38.18
1041	Z-ocimene	MH12	M	M	M	M	M	0.65	M	M	M	M	M	M	M	M	M	M	M	/
1056	E-ocimene	MH13	6.10	3.71	6.55	4.92	31.41	25.20	7.71	6.35	3.37	8.97	1.95	8.33	8.51	22.09	25.44	4.22	7.30	1.76
1068	γ-terpinene	MH14	2.69	19.12	1.59	1.70	M	M	27.07	5.88	M	3.09	7.38	2.63	M	M	M	26.66	4.25	M
1084	β-cymene	MH15	/	T	/	/	/	/	T	/	T	/	/	/	/	M	M	/	/	T
1093	Terpinolene	MH16	1.17	3.04	1.59	1.57	M	M	2.12	1.48	M	1.80	/	M	2.16	M	M	1.48	2.53	M
1113	1,3,8-*p*-menthatriene	MH17	/	/	/	/	T	T	/	/	T	/	T	T	T	T	M	/	/	T
1131	α-pyronene *	MH18	/	M	/	M	/	/	/	M	M	/	M	M	/	1.14	/	/	M	M
1143	Allo-ocimene	MH19	/	/	T	T	/	1.12	T	T	/	T	/	/	/	/	T	M	/	/
**Oxygenated monoterpenes**																				
1069	Terpineol, *cis*- β *	OM1	1.79	M	2.51	1.91	M	M	/	1.22	T	M	T	M	M	/	/	T	M	T
1112	Linalool	OM2	47.22	15.20	24.08	24.37	1.87	M	14.66	36.31	7.04	19.49	2.44	1.90	6.31	M	M	M	13.77	4.21
1117	Thujone *	OM3	/	/	/	/	/	/	/	T	T	/	T	M	M	/	/	/	T	T
1136	Limonene oxide, *cis* *	OM4	/	/	T	T	/	/	/	T	T	/	/	/	/	0.11	/	/	/	/
1139	Limonene oxide, *trans* *	OM5	/	/	T	T	/	/	T	/	M	/	/	M	M	/	/	/	M	M
1145	Citronellal	OM6	T	T	M	1.48	M	M	T	1.39	5.02	M	1.98	5.45	M	2.09	M	T	4.39	18.42
1148	Isopulegol	OM7	/	/	/	/	/	T	/	/	/	T	/	/	/	/	1.24	/	/	M
1156	Pinocarvone *	OM8	/	/	/	/	/	T	T	/	/	T	/	/	T	T	M	1.31	/	/
1161	Terpinen-4-ol	OM9	M	M	M	/	M	M	M	M	/	M	/	M	/	/	/	/	M	/
1177	Umbellulone *	OM10	T	/	/	M	/	/	/	T	/	/	/	/	M	/	M	/	/	/
1188	*p*-cymen-8-ol *	OM11	T	M	/	/	/	/	/	/	/	T	1.41	/	/	T	/	/	/	/
1192	α-terpineol	OM12	1.65	2.23	3.62	2.68	M	3.34	M	1.98	1.36	T	M	T	M	/	/	/	T	M
1198	*p*-menth-8-en-2-one *	OM13	/	/	T	T	/	/	M	/	T	/	/	T	/	/	/	/	T	T
1207	Carveol *	OM14	/	/	/	T	/	/	/	/	/	/	/	/	/	/	/	/	/	/
1216	*p*-menth-1-en-9-al *	OM15	/	/	/	T	/	/	/	T	M	/	/	/	T	/	/	/	T	M
1220	*cis*-carveol *	OM16	T	T	/	/	/	/	T	/	T	/	/	/	/	/	/	/	T	/
1231	*cis*-geraniol	OM17	T	T	M	M	/	M	T	M	M	T	1.22	M	M	/	T	/	4.60	M
1237	β-citronellol	OM18	/	/	T	M	/	/	/	M	/	/	/	M	M	/	/	/	/	M
1239	methyl thymyl ether	OM19	2.01	/	/	/	/	/	/	/	/	5.51	/	/	/	/	/	/	/	/
1243	β-citral	OM20	T	T	6.52	5.99	2.14	4.10	T	3.28	7.61	/	15.10	3.15	9.57	M	1.37	/	M	4.03
1252	*p*-menth-1-en-3-one	OM21	/	T	M	M	/	/	T	M	T	/	T	T	T	/	/	/	T	T
1258	*trans*-geraniol	OM22	T	T	M	M	T	T	T	M	M	T	M	T	M	T	T	/	T	M
1268	α-citral	OM23	T	T	8.93	8.92	2.93	5.42	M	4.81	11.23	T	19.65	4.06	12.47	1.02	1.32	/	M	5.32
1285	α-thujenal *	OM24	T	/	/	T	/	/	/	T	/	T	/	/	/	/	/	/	T	/
1300	p-Mentha-1(7),8(10)-dien-9-ol	OM25	M	/	T	T	/	/	T	1.37	M	T	T	/	T	/	/	T	M	T
1305	Carvacrol	OM26	T	/	/	/	/	/	T	T	/	/	/	/	/	/	/	T	/	/
1755	E,E-farnesal	OM27	/	/	/	T	/	/	T	/	T	T	/	T	T	/	/	/	/	T
**Sesquiterpene hydrocarbons**																				
1335	Ylangene *	SH1	/	/	T	/	/	/	/	/	M	/	/	M	/	1.92	/	/	/	M
1336	δ-elemene	SH2	1.64	M	/	/	/	/	1.34	/	/	3.79	/	/	T	/	/	2.37	/	/
1347	α-cubebene	SH3	T	M	T	T	M	/	M	T	T	T	T	T	/	/	/	M	T	T
1367	Copaene	SH4	T	/	/	/	/	/	T	T	/	M	/	/	/	/	/	T	/	/
1379	β-bourbonene	SH5	/	T	/	/	T	/	/	/	/	T	/	/	/	T	/	T	/	/
1393	β-elemene	SH6	/	12.93	4.88	6.42	M	M	7.04	2.79	8.76	/	7.80	M	6.41	M	1.37	10.63	6.31	/
1409	Zingiberene *	SH7	M	/	M	M	/	/	T	T	T	M	/	M	T	/	/	M	M	T
1415	Caryophyllene	SH8	1.07	6.50	1.65	2.27	5.26	2.54	M	1.90	5.23	1.79	4.76	1.48	1.83	2.74	T	M	2.13	4.43
1419	(*Z*)-α-bergamotene *	SH9	/	/	T	T	/	/	4.13	T	T	/	T	/	T	M	/	5.72	/	T
1423	β-cubebene	SH10	M	T	T	T	T	T	T	T	M	T	M	M	T	M	M	T		
1426	α-elemene *	SH11	T	/	/	/	/	/	/	T	/	/	/	T	/	/	/	/	T	/
1431	Aromadendrene	SH12	/	M	T	T	/	M	M	T	/	/	T	M	T	M	M	M	T	/
1433	γ-elemene*	SH13	2.66	T	/	/	1.93	1.06	2.39	/	T	5.28	/	/	/	2.05	M	3.20	M	T
1435	α-guaiene *	SH14	T	T	/	/	/	/	T	/	1.43	T	M	T	/	/	/	T	/	M
1441	α-himachalene *	SH15	/	/	T	T	M	/	/	/	/	/	/	/	/	/	/	T	/	/
1450	α-caryophyllene	SH16	M	1.67	M	M	M	M	1.14	M	1.19	M	M	M	M	M	M	1.64	M	M
1452	Allo-aromadendrene	SH17	T	/	/	/	/	/	/	/	/	T	T	T	/	T	T	/	/	/
1456	Bicyclosesquiphellandrene *	SH18	/	T	M	/	T	/	M	T	/	M	/	M	T	T	/	M	M	/
1457	β-santalene *	SH19	/	/	/	/	/	/	/	/	M	/	T	/	/	/	/	/	/	T
1462	β-farnesene	SH20	/	/	2.50	1.41	/	/	2.01	2.21	1.29	/	M	3.71	M	/	/	2.62	2.54	M
1470	γ-selinene *	SH21	2.15	M	/	/	/	/	/	/	T	2.90	T	T	/	/	/	/	/	T
1475	Germacrene D	SH22	M	/	T	T	M	M	/	T	M	M	T	/	T	M	T	1.75	T	M
1478	β-selinene	SH23	T	M	M	M	T	/	M	M	M	T	M	T	T	/	/	M	M	T
1481	γ-himachalene	SH24	/	T	T	T	/	/	T	T	T	/	T	/	T	/	/	T	T	T
1488	α-selinene	SH25	/	M	M	M	/	/	M	M	M	/	M	1.11	M	/	/	M	M	M
1493	δ-guaiene *	SH26	/	/	/	/	T	/	/	/	/	/	/	/	/	T	T	/	/	/
1496	α-muurolene	SH27	T	T	T	/	T	T	T	T	/	M	T	T	/	M	M	M	T	/
1497	β-gurjunene	SH28	T	/	/	T	/	/	/	/	T	T	/	/	T	/	/	/	/	T
1500	α-bulnesene *	SH29	T	T	T	T	/	/	T	/	/	T	/	/	/	/	/	T	/	T
1501	β-cadinene	SH30	/	/	T	/	/	/	M	/	/	/	T	/	/	/	/	T	/	/
1502	*cis*-α-bisabolene *	SH31	/	M	/	/	/	/	/	/	M	/	/	/	/	/	/	/	/	T
1503	α-amorphene	SH32	/	/	/	/	M	M	/	/	/	/	/	/	/	M	3.98	/	/	/
1504	γ-muurolene	SH33	M	ND	T	T	/	/	T	T	/	M	M	T	T	/	/	M	T	/
1505	β-bisabolene	SH34	/	/	/	/	/	/	/	/	2.21	/	/	/	/	/	/	/	/	1.21
1506	α-farnesene*	SH35	M	4.46	M	M	M	/	/	1.26	/	M	M	M	M	T	T	/	M	/
1509	δ-cadinene	SH36	M	M	T	T	M	/	/	T	M	M	M	M	T	/	M	M	M	T
1510	β-sesquiphellandrene	SH37	M	/	M	M	/	/	M	M	T	M	/	M	T	/	/	M	M	/
1512	8-isopropenyl-1,5-dimethyl-cyclodeca-1,5-diene *	SH38	/	/	/	/	T	T	M	/	/	/	/	/	/	T	T	T	/	/
1514	Valencene	SH39	/	/	/	/	/	/	T	/	T	/	/	/	/	/	/	T	/	T
1515	Eudesma-3,7(11)-diene *	SH40	T	T	/	T	T	/	T	/	/	M	/	T	T	T	/	M	/	/
1526	α-panasinsene *	SH41	/	/	/	/	/	/	/	/	/	T	/	/	/	/	/	/	/	/
1531	(E,Z)-α-farnesene	SH42	/	/	/	/	/	/	/	/	/	/	/	/	/	/	/	/	/	/
1643	Cadalene *	SH43	/	/	/	T	/	/	T	/	T	/	/	/	T	/	/	/	/	/
**Oxygenated sesquiterpenes**																				
1530	Nerolidol 2	OS1	T	/	/	/	/	M	T	T	T	T	/	/	/	/	T	/	T	T
1531	Germacrene D-4-ol	OS2	T	T	/	/	M	M	/	/	/	T	/	T	/	/	M	/	/	/
1587	Caryophyllene oxide	OS3	T	M	/	T	T	T	T	T	T	T	T	T	T	T	/	/	T	T
1592	α-humulene oxide *	OS4	/	/	/	/	/	/	/	/	T	/	/	/	/	/	/	/	/	/
1614	Cubenol *	OS5	T	T	/	/	T	/	T	/	T	T	/	/	/	/	/	/	/	/
1626	Spathulenol	OS6	T	/	/	/	/	/	T	/	/	T	/	/	/	/	/	T	/	/
1657	β-eudesmol	OS7	T	/	/	/	/	M	T	/	/	T	/	/	/	/	T	/	T	/
1765	α-sinensal	OS8	M	/	T	T	/	/	/	T	/	T	/	/	T	/	/	/	/	/
**Ketones**																				
1399	*cis*-jasmone *	K1	/	T	/	/	/	/	/	T	T	/	/	/	/	/	/	/	T	/
1484	β-ionone *	K2	/	/	T	T	T	T	/	T	/	/	/	/	/	/	/	/	T	T
**Esters**																				
1219	Acetic acid, octyl ester *	E1	/	/	/	/	/	/	/	/	/	/	/	/	/	/	/	/	T	/
1328	Methyl geranate	E2	/	/	T	T	/	/	/	T	/	/	T	T	T	/	/	/	M	/
1330	Myrtenyl acetate *	E3	T	/	/	/	/	/	/	/	/	T	/	/	T	/	/	/	/	/
1360	Citronellol acetate	E4	T	/	/	T	/	/	T	/	T	T	M	M	2.66	M	/	T	M	1.24
1370	Nerol acetate	E5	/	/	T	M	/	M	/	/	M	/	3.93	M	4.49	M	2.39	/	1.63	2.21
1398	Geranyl acetate	E6	M	/	T	T	/	/	/	/	/	1.34	/	/	/	/	/	/	/	5.25
**Miscellaneous**																				
877	Styrene *	Ah1	/	T	T	T	/	/	T	/	T	/	/	/	/	M	T	/	T	T
1176	Naphthalene *	Ah2	/	/	/	/	/	/	/	/	M	/	/	/	/	T	T	T	/	M
966	Phenol *	P1	/	/	/	/	/	/	/	/	/	/	/	/	/	/	1.56	/	/	/
1298	*p*-thymol *	P2	/	M	/	/	/	/	T	/	/	/	T	/	/	/	/	/	/	/
1076	1-octanol	Ac1	/	/	/	/	/	M	/	/	/	/	/	/	/	T	/	T	/	/
1292	Phenyl-but-3-en-1-ol *	Ac2	T	/	T	T	/	/	/	T	/	T	/	T	/	/	/	/	/	/
1172	Vinylcyclohexane *	U1	/	/	/	M	/	/	/	M	M	/	/	/	M	/	/	/	T	/
1387	1-decen-3-yne *	U2	/	/	/	/	/	/	/	T	/	/	/	/	T	/	/	/	T	/
1286	Indole	ND	/	T	/	/	/	/	T	/	M	/	/	/	/	/	/	/	/	/

Data are arranged according to chemical groups and represent the mean percentage of individual leaf constituents from triplicate experiments; letter indicated by: T, Trace (<0.1%); M, moderate (between 0.1% and <1%).

a Retention indices; analyzed on HP-5MS column;

b Compounds marked with * indicate those reported for the first time in citrus leaves;

c Family code. A, aldehyde; MH, monoterpene hydrocarbons; OM, oxygenated monoterpenes; SH, sesquiterpene hydrocarbons; OS, oxygenated sesquiterpenes; Km ketones; E, esters; Ah, aromatic hydrocarbons; P, phenolic; Ac, alcohol; U, unclassified; ND, nitrogen derivative.

d The full cultivar names corresponding to the abbreviations are the same as indicated in Table 1;

e Undetectable.

**Table 3:** Major volatiles compounds, in percentages and rank, of young leaves from nine citrus cultivars.

Compounds	FC a	PK b	ST	LBC	QJ	YHY	ZXY	HY	HSG	ERK	Mean rank
Linalool	OM2	47.22 ^1st^	15.20 ^2nd^	24.08 ^1st^	24.37 ^1st^	1.87 ^11th^	0.15 ^33th^	14.66 ^2nd^	36.31 ^1st^	7.04 ^5th^	18.99 ^1st^
E-ocimene	MH13	6.10 ^3rd^	3.71 ^8th^	6.5 ^4th^	4.92 ^6th^	31.41 ^1st^	25.20 ^1st^	7.71 ^5th^	6.35 ^3th^	3.37 ^8th^	10.59 ^2nd^
β-pinene	MH4	/c	5.97 ^6th^	/	/	27.91 ^2nd^	21.32 ^2nd^	10.18 ^3rd^	11.23 ^2nd^	1.04 ^16th^	8.63 ^3rd^
Limonene	MH11	2.99 ^4th^	/	4.34 ^7th^	4.10 ^7th^	3.64 ^6th^	4.60 ^5th^	7.91 ^4th^	4.63 ^6th^	31.71 ^1st^	7.10 ^4th^
γ-terpinene	MH14	2.69 ^6th^	19.12 ^1st^	1.59 ^15th^	1.70 ^14th^	0.37 ^20th^	0.55 ^18th^	27.07 ^1st^	5.88 ^4th^	0.73 ^18th^	6.63 ^5th^
β-terpinene	MH5	14.38 ^2nd^	/	15.18 ^2nd^	15.36 ^2nd^	4.04 ^5th^	/	/	1.65 ^14th^	/	5.62 ^6th^
β-elemene	SH6	/	12.93 ^4th^	4.88 ^6th^	6.42 ^4th^	0.32 ^22th^	0.15 ^5th^	7.04 ^6th^	2.79 ^8th^	8.76 ^3rd^	4.81 ^7th^
α-Citral	OM23	0.04 ^45th^	0.02 ^50th^	8.93 ^3rd^	8.92 ^3rd^	2.93 ^7th^	5.42 ^4th^	0.11 ^32th^	4.81 ^5th^	11.23 ^2nd^	4.71 ^8th^
β-Citral	OM20	0.03 ^53th^	0.01 ^55th^	6.52 ^5th^	5.99 ^5th^	2.14 ^9th^	4.10 ^7th^	0.08 ^38th^	3.28 ^7th^	7.61 ^4th^	3.31 ^9th^
2-hexenal	A2	0.67 ^19th^	1.31 ^13th^	0.92 ^17th^	0.83 ^19th^	7.15 ^3rd^	13.64 ^3rd^	1.74 ^12th^	1.17 ^20th^	1.20 ^14th^	3.18 ^10th^
Caryophyllene	SH8	1.07 ^15th^	6.50 ^5th^	1.65 ^14th^	2.27 ^11th^	5.26 ^4th^	2.54 ^10th^	0.12 ^31th^	1.90 ^12th^	5.23 ^6th^	2.95 ^11th^
α-terpineol	OM12	1.65 ^12th^	2.23 ^10th^	3.62 ^9th^	2.68 ^10th^	0.83 ^13th^	3.34 ^8th^	0.26 ^23th^	1.98 ^11th^	1.36 ^9th^	2.00 ^12th^
*p*-cymene	MH10	/	14.35 ^3rd^	/	/	/	/	/	/	/	1.59 ^13th^
α-thujene	MH1	2.70 ^5th^	0.44 ^20th^	2.58 ^10th^	3.27 ^9th^	0.48 ^18th^	0.73 ^15th^	0.76 ^17th^	2.24 ^9th^	0.05 ^48th^	1.47 ^14th^
Terpinolene	MH16	1.17 ^14th^	3.04 ^9th^	1.59 ^16th^	1.57 ^15th^	0.27 ^25th^	0.39 ^22th^	2.12 ^10th^	1.48 ^15th^	0.21 ^16th^	1.31 ^15th^
Citronellal	OM6	0.07 ^14th^	0.03 ^14th^	0.85 ^17th^	1.48 ^15th^	0.21 ^16th^	0.47 ^12th^	0.04 ^17th^	1.39 ^16th^	5.02 ^7th^	1.06 ^16th^
β-myrcene	MH6	/	/	2.01 ^13th^	2.17 ^12th^	/	0.47 ^20th^	1.01 ^15th^	/	1.22 ^13th^	1.07 ^17th^
3-carene	MH8	/	/	4.23 ^8th^	3.31 ^8th^	/	/	/	/	/	1.05 ^18th^
β-farnesene	SH19	/	/	2.50 ^12th^	1.41 ^17th^	/	/	2.01 ^11th^	/	1.29 ^12th^	1.05 ^19th^
α-pinene	MH2	/	1.32 ^12th^	/	/	2.56 ^8th^	1.96 ^11th^	2.24 ^9th^	/	0.19 ^33th^	0.92 ^20th^

a Family code;

b The full names corresponding to the abbreviations are as indicated in Table 1;

c Undetectable.

**Table 4:** Major volatiles compounds, in percentages and rank, of mature leaves from nine citrus cultivars.

Compounds	FC a	PK b	ST	LBC	QJ	YHY	ZXY	HY	HSG	ERK	Mean rank
Limonene	MH11	4.11 ^7th^	13.71 ^3rd^	13.06 ^2th^	7.29 ^6th^	6.93 ^4th^	4.47 ^5th^	7.40 ^5th^	7.78 ^3rd^	38.18 ^1st^	11.44 ^1st^
E-ocimene	MH13	8.97 ^3rd^	1.95 ^11th^	8.33 ^3th^	8.51 ^5th^	22.09 ^1st^	25.44 ^1st^	4.22 ^7th^	7.30 ^4th^	1.76 ^10th^	9.84 ^2nd^
β-pinene	MH4	/c	7.29 ^6th^	/	/	21.88 ^2nd^	20.65 ^2nd^	11.11 ^2nd^	18.05 ^1st^	1.06 ^14th^	8.89 ^3rd^
β-terpinene	MH5	21.83 ^1st^	/	30.55 ^1st^	13.64 ^1st^	5.70 ^6th^	/	/	3.38 ^11th^	/	8.34 ^4th^
Linalool	OM2	19.49 ^2nd^	2.44 ^9th^	1.90 ^12th^	6.31 ^8th^	0.15^31th^	0.31 ^24th^	0.41 ^21th^	13.77 ^2nd^	4.21 ^6th^	5.44 ^5th^
2-hexenal	A2	2.21 ^12th^	1.37 ^13th^	2.63 ^10th^	1.25 ^15th^	14.29 ^3rd^	14.60 ^3rd^	7.83 ^4th^	2.23 ^14th^	1.72 ^11th^	5.35 ^6th^
γ-terpinene	MH14	3.09 ^8th^	7.38 ^5th^	2.63 ^11th^	0.96 ^16th^	0.48 ^19th^	0.56 ^19th^	26.66 ^1st^	4.25 ^9th^	0.23 ^27th^	5.14 ^7th^
α-citral	OM23	0.01 ^13th^	19.65 ^1st^	4.06 ^7th^	12.47 ^2nd^	1.02 ^13th^	1.32 ^14th^	/	0.86 ^19th^	5.32 ^3rd^	4.97 ^8th^
β-citral	OM20	/	15.10 ^2nd^	3.15 ^9th^	9.57 ^3rd^	0.78 ^16th^	1.37 ^13th^	/	0.67 ^22th^	/	3.85 ^9th^
β-elemene	SH6	/	7.80 ^4th^	0.23 ^27th^	6.41 ^7th^	0.85 ^14th^	1.37 ^12th^	10.63 ^3rd^	6.31 ^5th^	/	3.73 ^10th^
Citronellal	OM6	0.13 ^12th^	1.98 ^10th^	5.45 ^5th^	0.82 ^18th^	2.09 ^9th^	0.18 ^27th^	0.02 ^15th^	4.39 ^8th^	18.42 ^2nd^	3.72 ^11th^
α-thujene	MH1	5.27 ^6th^	0.37 ^24th^	7.95 ^4th^	2.26 ^12th^	0.62 ^18th^	0.75 ^18th^	1.03 ^17th^	3.70 ^10th^	/	2.44 ^12th^
Hexanal	A1	0.84 ^17th^	0.29 ^26th^	0.82 ^17th^	0.25 ^28th^	6.86 ^5th^	7.05 ^4th^	2.67 ^9th^	0.87 ^18th^	0.23 ^26th^	2.21 ^13th^
Caryophyllene	SH8	1.79 ^14th^	4.76 ^7th^	1.48 ^14th^	1.83 ^14th^	2.74 ^7th^	0.07 ^17th^	0.22 ^24th^	2.13 ^15th^	4.43 ^5th^	2.16 ^14th^
β-myrcene	MH6	3.50 ^9th^	0.96 ^17th^	4.74 ^6th^	3.46 ^10th^	/	1.41 ^11th^	0.95 ^19th^	/	1.80 ^9th^	1.87 ^15th^
3-carene	MH8	/	0.96 ^16th^	/	8.51 ^4th^	/	/	/	5.87^6th^	/	1.70^16th^
Nerol acetate	E5	/	3.93 ^8th^	0.23 ^25th^	4.49 ^9th^	0.42 ^20th^	2.39^9th^	/	1.63^16th^	2.21^8th^	1.70^17th^
γ-elemene	SH13	5.28 ^6th^	/	/	/	2.05^10th^	0.79^16th^	3.20^8th^	0.17^30th^	0.03^14th^	1.28^18th^
β-farnesene	SH19	/	0.12 ^17th^	/	/	/	/	2.62^10th^	2.54^12th^	0.25^25th^	1.08^19th^
α-pinene	MH2	/	1.33 ^14th^	/	/	2.10^8th^	3.11^7th^	2.56^11th^	/	0.39^23th^	1.06^20th^

a Family code;

b The full names corresponding to the abbreviations are as indicated in Table 1;

c Undetectable.

## Supplementary Files

Supplementary File 1 Supplementarya (ZIP, 2135 KB)
