# Supplementary material for: Citrus Leaf Volatiles as Affected by Developmental Stage and Genetic Type
Source: Int J Mol Sci. 2013 Aug 29;14(9):17744–66. doi: 10.3390/ijms140917744 (PMC3794751; doi:10.3390/ijms140917744)
Supplement: Supplementary File 1 — Supplementarya (ZIP, 2135 KB) [file ijms-14-17744-s001.zip › ijms-32595-final-suppl/ijms-32595-Figure S1ú¡proofed without change.doc]

**Figure S1.** Chromatogram of E-Nose and gas chromatography-mass spectrometry (GC-MS) of young (Y) and mature (M) leaf from nine citrus cultivars.
